# Stress, Depression, and Unhealthy Behavior Changes among Patients with Diabetes during COVID-19 in Korea

**DOI:** 10.3390/healthcare10020303

**Published:** 2022-02-04

**Authors:** Hae Ran Kim, Jeong-Soon Kim

**Affiliations:** 1 Department of Nursing, College of Medicine, Chosun University, Gwangju 61452, Korea; rahn00@chosun.ac.kr; 2 Department of Nursing, Gwangju Health University, Gwangju 62287, Korea

**Keywords:** diabetes, COVID-19, depression, stress, health behavior, health surveys

## Abstract

The government ordered various restrictions to limit the spread of coronavirus disease 2019 (COVID-19), thus, affecting the mental health status and lifestyle of people with diabetes. This study identifies COVID-19 effects on mental health problems and unhealthy behavioral changes among patients with diabetes. The subjects of this cross-sectional study were adults aged 19 years or older who participated in the 2020 Korean Community Health Survey. Stress, depression, and changes in unhealthy behavior in diabetic patients (N = 26,839) because of COVID-19 were compared with controls (N = 26,834). The association between stress and depression and unhealthy behaviors among patients with diabetes was investigated. During the COVID-19 pandemic, 20.3% and 4.2% of diabetic patients reported higher levels of stress and depression, respectively, than controls. Diabetic patients showed decreased physical activity and sleep time, and increased smoking. Among diabetic patients, stress and depression are associated with unhealthy behavior changes during COVID-19. Measures to promote healthy lifestyles along with stress and depression management strategies must be implemented for the health care of diabetic patients during the pandemic.

## 1. Introduction

Coronavirus disease 2019 (COVID-19), first reported in November 2019, has created a global public health emergency. As of September 2021, the virus has infected over 267,000 people and caused over 2000 deaths in South Korea [1]. The South Korean government ordered social distancing, travel restrictions, and a ban on largescale events to prevent the spread of the infection—affecting the daily lifestyle of the public [2].

Although new literature on COVID-19 has been growing rapidly in recent years, little is known about the impact of the psychological burden on people with diabetes during the pandemic. In previous studies, infectious diseases caused psychological distress in various countries due to prognostic uncertainty and social isolation to prevent infection [3]. COVID-19 increases mental health problems, such as anxiety, depression, and stress [4,5]. In particular, COVID-19 has caused psychological distress due to stress and depression in people with chronic diseases such as diabetes [6]. Currently, the increasing psychological burden from the effects of the COVID-19 pandemic on people with diabetes may affect their health behaviors [7,8]. Diabetes is a chronic disease in which self-management is very important. Various psychosocial problems of diabetic patients have an important effect on the self-management of diabetes [9]. Depression is a very important psychological and social factor that influences diabetes management [10].

Prior to the pandemic, people with diabetes were more likely to report stress and depression than those without [11,12,13]. COVID-19 may exacerbate stress and depression in people with diabetes, which is the most reported comorbidity in COVID-19 patients [9]; it causes several psychosocial problems [12], and restricted social activities to prevent viral infection may make psychological adjustment difficult [14]. According to a recent study, during the COVID-19 pandemic, patients with diabetes reported higher levels of psychological stress and depression compared to those without diabetes because of fear of infection, concerns about lack of treatment drugs, and risk of symptoms requiring hospitalization [8]. Mental health problems may affect cortisol dysregulation, exacerbating diabetes during the COVID-19 pandemic [15].

Despite worsening glycemic control, people with diabetes reported decreased sleep duration and physical activity, increased unhealthy eating habits, drinking, and smoking during the pandemic [16,17,18]. According to previous studies, unhealthy behaviors in diabetic patients may be related to mental health problems. For example, psychological stress over the risk of infection may affect sleeping habits [18]. Some people may rely on fast food consumption, alcohol use, and smoking to cope with negative emotions [19,20]. Therefore, investigating mental health and unhealthy behavior changes in diabetes during the COVID-19 pandemic may help promote positive self-management in diabetic patients.

Currently, studies on the psychological distress and unhealthy behavior of patients with diabetes during the COVID-19 pandemic are limited. Early evidence during the COVID-19 outbreak suggests a link between mental health problems and unhealthy behavioral changes [21]. This study compared stress, depression, and unhealthy behavioral changes between patients with diabetes and controls during the COVID-19 pandemic using the 2020 Korean Community Health Survey (KCHS, 2020). It also examined the association between stress, depression, and unhealthy behavior changes among patients with diabetes.

## 2. Methods

### 2.1. Data Source and Study Participants

This study utilized data from the KCHS, conducted from August 16 to October 31, 2020, by the Korea Disease Control and Prevention Agency (KCDA). The KCHS is a nationwide health survey of adults aged 19 years and older that has been conducted since 2008. The KCHS survey is based on a two-stage stratified sample of households. Trained researchers interviewed participants using a computer assisted personal interviewing process [22]. The Institutional Review Board of the KCDA approved the survey protocol (2016-10-01-P-A).

The KCHS data used in this study included 142 questions across 18 fields, including health behavior, physical activity, medical services use, and social environment. There was a total of 229,269 participants. Those who answered “yes” to the question, “Have you ever been told by a doctor that you had diabetes?” were classified as having diabetes. The participants were asked about their awareness of glucose level, age at diagnosis, duration of diabetes, and diabetes treatment. Of the total respondents aged ≥19 years, 26,839 were diagnosed with diabetes. Control subjects were defined as people who reported no history of diabetes. Sex and age matched controls (1:1) were selected from the non-diabetic subjects. Sex and age are known to be the most important confounding variables [23]. Finally, 26,839 diabetes and 26,834 controls were selected.

### 2.2. Variables

Mental health included self-rated perceived stress and depression. Perceived stress was defined as a “very much” or “much” answer to the question, “How much do you feel stressed in everyday life?” Depression was assessed using the Patient Health Questionnaire-9 (PHQ-9). The PHQ-9 items for each component were scored on a 4-point Likert scale ranging from 0 to 27. Scores greater than 10 were classified as depressed [24].

Unhealthy behavior changes because of COVID-19 included changes in physical activity such as walking and exercise (including both indoor and outdoor), changes in sleep time, changes in junk food or carbonated beverages consumption, changes in food delivery consumption, changes in alcohol consumption, and changes in smoking behavior. Participants were asked about the change in their health behavior with the following question: “Compared to pre-COVID-19, has your physical activity such as walking and exercise (including both indoor and outdoor) changed?” “Compared to pre-COVID-19, has your sleep time changed?” “Compared to pre-COVID-19, has your junk food or carbonated beverages consumption changed?” “Compared to pre-COVID-19, has your food delivery consumption changed?” “Compared to pre-COVID-19, has your alcohol consumption changed?” and “Compared to pre-COVID-19, has your smoking behavior changed?” Response options included: “in-creased/no-changed/decreased.” Unhealthy behavior changes were classified: responded with decreased changes in physical activity and sleep time and responded with increased changes in junk food or carbonated beverages consumption, food delivery consumption, alcohol consumption, and smoking behavior.

### 2.3. Covariates

The covariates included were sex (male or female), age (19–44, 45–64, 65–74 ≥ 75), education level (≤middle school, high school, ≥college), family (living alone, living with a spouse, others), monthly income (high, middle-high, middle-low, low), location of residence (urban or rural), hypertension diagnosis (yes or no), subjective health status (good or poor), current smoker (yes or no), and current alcohol use (yes, no). Hypertension was defined based on the diagnoses by physicians.

In diabetic patients, the following diabetes-related characteristics were also assessed: awareness of glucose level (yes, no), age at diagnosis, duration of diabetes, and diabetes treatment (no, yes; non-therapy (including exercise and diet), an oral hypoglycemic agent, insulin injection, and both oral agents and insulin).

### 2.4. Statistical Analysis

The general characteristics of diabetes and control subjects are presented as numbers and percentages. Chi-square tests were used to analyze the differences in general characteristics between the groups. Mental health status and unhealthy behavior changes between patients with diabetes and controls were analyzed using chi-square tests and multiple logistic regressions. Multiple logistic regression was used to analyze the association between mental health status and unhealthy behavioral changes among patients with diabetes. SAS version 9.4 (SAS Institute, Cary, NC, USA) survey procedure was used to account for the complex sampling design, with *p* < 0.05, considered statistically significant.

## 3. Results

Family type and location of residence were not significantly different between diabetes and control groups. Poor subjective health status and current smoking rates were higher, and the current alcohol user rate was lower in patients with diabetes than in controls. Of the patients, 57.5% had diabetes for over 5 years from their date of diabetes diagnosis, and the most common treatment was oral agents (Table 1).

Perceived stress and depression were reported in 20.3% and 4.2% of patients with diabetes, respectively. Among patients with diabetes, 41.8% and 8.6% reported decreased changes in physical activity and sleep time, and 10.8%, 24.4%, 7.6%, and 9.5% reported increased changes in junk food or carbonated beverage consumption, food delivery consumption, alcohol consumption, and smoking behavior because of COVID-19, respectively. The proportion of decreased change in physical activity (*p* = 0.012), sleep time (*p* = 0.005), and proportion of increased change in smoking behavior (*p* = 0.001) in diabetes were significantly higher than in controls. The proportion of increased change in food delivery consumption in diabetes patients was lower than that in controls (*p* = 0.043). Diabetes was more likely to report perceived stress (adjusted odds ratio [OR] = 1.13, 95% confidence interval [CI] = 1.08–1.18), depression (aOR = 1.21, 95% CI = 1.09–1.34), and increased smoking behavior (aOR = 1.19, 95% CI = 1.01–1.41) than controls (Table 2).

Among diabetes patients, participants who reported perceived stress were more likely to have decreased change in physical activity (aOR = 1.35, 95% CI = 1.26–1.45) and sleep time (aOR = 2.21, 95% CI = 2.00–2.44), and increased change in junk food or carbonated beverages consumption (aOR = 1.82, 95% CI = 1.58–2.09), food delivery (aOR = 1.50, 95% CI = 1.32–1.70), alcohol consumption (aOR = 2.22, 95% CI = 1.80–2.72), and smoking behavior (aOR = 2.17, 95% CI = 1.75–2.70). Diabetes patients who reported depression were more likely to have decreased change in physical activity (aOR = 1.34, 95% CI = 1.15–1.55) and sleep time (aOR = 1.87, 95% CI = 1.56–2.24), and increased change in junk food or carbonated beverages consumption (aOR = 1.48, 95% CI = 1.11–1.99), food delivery (aOR = 1.54, 95% CI = 1.15–2.08), alcohol consumption (aOR = 2.46, 95% CI = 1.62–3.71), and smoking behavior (aOR = 1.92, 95% CI = 1.27–2.90) (Table 3).

## 4. Discussion

In this study, stress, depression, and unhealthy behavior changes in diabetic patients during COVID-19 were compared to controls, and the association between mental health problems and unhealthy behavior changes among diabetic patients was investigated. According to this study, Korean adults with diabetes had a higher risk of stress and depression than adults without diabetes during the pandemic. Among diabetic patients, stress and depression are associated with unhealthy behavioral changes.

Compared to previous studies, the prevalence of stress (20.3%) in this study was lower. In India, 87% of diabetics reported stress due to the lockdown, and more than 80% of diabetics in the US reported higher stress post COVID-19 [25,26]. Compared to previous studies conducted in lockdown situations or online surveys, this study collected data through face-to-face direct surveys, and South Korea never imposed a full lockdown. This may explain the low prevalence of stress in the country. Depression has been reported in 4.2% of patients with diabetes. Depression prevalence was lower than that reported by 60.7% of diabetics in the Arab Gulf Region [8], and 23.1% of diabetics in Saudi Arabia during the COVID-19 [17]. There are several possible explanations for these results. First, it may be because of methodological differences in sampling and recruitment strategies. For example, an online survey was used to collect data from previous studies, and in this study, data were collected face-to-face with the population according to national policy. Social desirability bias during face-to-face surveys may cause some participants to under-report depression [27]. Second, there may be differences in prevalence according to the depression definition by the PHQ-9. In a previous study, a score of 5 or higher was defined as depression, and in this study, a score of 10 or higher was defined as depression as a diagnostic criterion for depressive disorder [24]. This may have contributed to the lower prevalence of depression in diabetic patients. Third, since the data were collected in Korea between August and October 2020, this could explain the difference in the timing of data collection, the difference in the government response to the pandemic, and the severity of the impact on the population.

As before the pandemic, during the COVID-19 pandemic, diabetic patients had a higher incidence of stress and depression compared to controls, and depression could be a risk factor for suicide in diabetic patients [28]. During the pandemic, the psychological problems of diabetic patients are often unrecognized or underestimated, which may impair quality of life and self-management for many diabetic patients [29]. It has been reported that stress and depression are maintained from the beginning to the end of lockdown because of the COVID-19 [30]. During the coronavirus, diabetics may be more susceptible to depression than people without diabetes [5], therefore, psychological problems may persist for longer. These results suggest that careful monitoring of mental health problems in patients with diabetes is important during a pandemic.

A total of 41.8%, 8.6%, and 9.5% of diabetic patients reported decreased physical activity and sleep time, respectively—significantly higher than that of the controls. Reduced physical activity has been reported worldwide because of movement restrictions to prevent the spread of infection [31]. A recent Korean study reported an association between reduced physical activity and weight gain and higher glucose levels in patients with diabetes [32]. Regular physical activity that can maintain fitness levels while maintaining social distance during epidemics can help with proper weight and good glycemic control in diabetic patients [33]. Insufficient sleep time during the pandemic may reflect anxiety associated with high concerns about the risk of contracting COVID-19 while having diabetes [34]. Sleep disturbance among diabetic patients may lead to elevated blood glucose levels and impair immune responses, leading to a poor prognosis when infected with SARS-CoV-2 [18]. For a better understanding, specific studies on sleep disturbance factors in diabetic patients are needed.

Similar to previous studies, the proportion of smokers with diabetes was significantly higher than that in controls [7], and 90.5% of diabetic patients did not change their smoking behavior after the pandemic. Since smokers are more susceptible to respiratory infections, smoking has been reported as a risk factor for COVID-19 severity and mortality in patients [9]. Health promotion efforts are needed to educate people with diabetes about the risks of smoking during the COVID-19 pandemic.

Additionally, 10.8%, 24.4%, and 7.6% of diabetic patients reported increased junk food or carbonated beverages, food delivery, and alcohol consumption, respectively. During the COVID-19 pandemic, social distancing has affected the increase in delivery of a variety of foods in South Korea, and in general, popular delivery foods were fast food, junk food, and coffee or beverages that are less fresh than home-made foods [35]. Diabetics with increased alcohol consumption during the pandemic were more likely to have worse glycemic control [16]. Obesity in diabetic patients because of these diets leads to detrimental consequences of COVID-19 infection [36]. During an epidemic, diabetics should be educated on healthy eating to help strengthen the immune system and reduce inflammation.

Among diabetic patients, stress and depression were significantly associated with decreased physical activity and sleep time, increased consumption of junk food or carbonated beverages, food delivery, alcohol consumption, and smoking because of COVID-19. These results are consistent with those of previous studies showing that psychological problems are more likely to catalyze negative health behaviors. For example, a recent meta-analysis found that stress and depression experienced during COVID-19 were significantly associated with decreased physical activity [4], and decreased mental well-being while working from home was associated with decreased physical activity and exercise and increased junk food intake [37]. A study published in Europe found that negative changes in mental health during lockdown were associated with decreased physical activity and increased sedentary behavior [38]. Diabetics who experience higher levels of psychological distress than the general population during a pandemic are more likely to participate in lower healthy behaviors [6]. These results suggest that monitoring and supporting the appropriate mental health care needs of diabetic patients is important in reducing unhealthy behaviors.

This study is the first published study to report an association between mental health problems and unhealthy behavior in Korean adult patients with diabetes during the COVID-19 pandemic. Numerous studies have highlighted the need for a rapid and comprehensive response to the growing mental health needs for the healthcare of diabetic patients during COVID-19 [21,29,30]. Given the scale of the COVID-19 pandemic, this support is expected to be maintained for years to come.

This study has some limitations. First, since this study included adults who were aware of diabetes, diabetes was evaluated based on self-reported measurements, not fasting blood glucose and glycated hemoglobin A1c (HbA1c) tests. Second, because of the cross-sectional nature of these data, stress, and depression before COVID-19 in diabetic patients could not be investigated. Stress and depression have been shown to increase the risk of incident type 2 diabetes mellitus [11]. Third, a self-report method was used to measure stress, depression, and unhealthy behaviors during COVID-19. A socially desirable bias may have influenced participants to under-report their responses. Finally, because the data are cross-sectional, causal relationships cannot be inferred. Longitudinal data are needed to observe changes over time to evaluate the impact of changes in social restrictions caused by a pandemic.

## 5. Conclusions

In conclusion, this study suggests that mental health problems in Korean patients with diabetes are associated with negative changes in health behavior because of the COVID-19 pandemic. The acute and chronic increase in mental health problems must be reduced during this unprecedented time using effective health promotion strategies to adopt or maintain positive health-related behaviors, such as social media messaging and remote management for people with diabetes. Ongoing assessment of the impact of the psychosocial environment associated with the pandemic on health behavior is necessary to develop strategies to promote a healthy lifestyle for diabetic patients.

## Figures and Tables

**Table 1:** Characteristics of diabetes and control subjects.

Characteristics	Non-Diabetes (N = 26,834)		Diabetes (N = 26,839)		*p*
	n	%	n	%	
Sex					
Male	13,214	49.2	13,218	49.2	1.000
Female	13,620	50.8	13,621	50.8	
Age					
19–44	1050	3.9	1050	3.9	1.000
45–64	9678	36.1	9679	36.1	
65–74	8596	32.0	8597	32.0	
75≤	7510	28.0	7513	28.0	
Education level					
≤Middle school	15,112	56.4	15,874	59.2	<0.001
High school	7023	26.2	7146	26.7	
≥College	4653	17.4	3784	14.1	
Family type					
Living alone	5498	20.5	5630	20.9	0.115
Living with spouse	11,602	43.2	11,691	43.6	
Others	9733	36.3	9517	35.5	
Monthly income (10,000 won)					
High	7012	26.1	6408	23.9	<0.001
Middle-high	3547	13.2	3278	12.2	
Middle-low	7854	29.3	8177	30.5	
Low	8421	31.4	8976	33.4	
Location of residence					
Urban (dong)	12,490	46.6	12,720	47.4	0.049
Rural (eup or myeon)	14,344	53.5	14,119	52.6	
Hypertension diagnosis					
No	16,366	61.4	10,270	38.3	<0.001
Yes	10,465	38.6	16,565	61.7	
Subjective health status					
Good	11,302	42.1	6746	25.1	<0.001
Poor	15,531	57.9	20,091	74.9	
Current smoker					
No	22,835	85.1	22,545	84.0	0.000
Yes	3994	14.9	4289	16.0	
Current alcohol user					
No	17,125	63.8	18,533	69.1	<0.001
Yes	9705	36.2	8305	30.9	
Awareness of glucose level					
Yes			20,588	76.9	
No			6202	23.1	
Age at diagnosis (years)					
≤19			80	0.3	
20–29			261	0.9	
30–39			1551	5.8	
40–49			4643	17.4	
50–59			8259	30.9	
60≤			11,969	44.7	
Duration of diabetes, n (%), years					
≤5 years			11,392	42.5	
>5 years			15,447	57.5	
Diabetes treatment					
No			807	3.0	
Yes			26,025	97.0	
Treatment method					
Non-drug therapy (exercise, diet)			9831	36.6	
Oral agent (oral hypoglycemic agent)			24,555	91.5	
Insulin injection			1963	7.3	
Oral agent and insulin injection			1640	6.1	

Data are expressed as number (%).

**Table 2:** Mental health status and unhealthy behavior changes due to COVID-19 among diabetes compared to controls.

Characteristics	Non-Diabetes		Diabetes		*p*
Mental Health Status	n	%	n	%	
Perceived stress ^a^					
No	22,361	83.4	21,372	79.7	<0.001
Yes	4463	16.6	5458	20.3	
aOR (95% CI) for perceived stress a	1.00		1.13 (1.08–1.18)		
Depression (PHQ-9)					
<10	25,991	97.4	25,576	95.8	<0.001
≥10	707	2.6	1119	4.2	
aOR (95% CI) for ≥10 ^b^	1.00		1.21 (1.09–1.34)		
**Unhealthy Behavior Changes due to COVID-19**					
Changes in physical activity such as walking and exercise (including both indoor and outdoor)					
No change	13,671	59.3	13,352	58.2	0.012
Decrease	9375	40.7	9599	41.8	
aOR (95% CI) for decrease ^a^	1.00		1.01 (0.97–1.05)		
Changes in sleep time					
No change	22,767	92.1	22,358	91.4	0.005
Decrease	1949	7.9	2099	8.6	
aOR (95% CI) for decrease ^a^	1.00		1.02 (0.95–1.09)		
Changes in consumption of junk food or carbonated beverages					
No change	9728	89.0	9228	89.2	0.770
Increase	1197	11.0	1121	10.8	
aOR (95% CI) for increase ^a^	1.00		1.00 (0.91–1.10)		
Changes in food delivery consumption					
No change	5958	74.2	5784	75.6	0.043
Increase	2073	25.8	1868	24.4	
aOR (95% CI) for increase ^a^	1.00		0.96 (0.89–1.05)		
Change in alcohol consumption					
No change	6698	92.3	5802	92.4	.770
Increase	560	7.7	476	7.6	
aOR (95% CI) for increase ^a^	1.00		0.97 (0.85–1.11)		
Change in smoking behavior					
No change	3895	92.5	4000	90.5	0.001
Increase	314	7.5	418	9.5	
aOR (95% CI) for increase ^a^	1.00		1.19 (1.01–1.41)		

COVID-19 = Coronavirus Disease-19; aOR, adjusted odds ratio; CI, confidence interval; ^a^ Adjusted for sex, age, educational level, family type, monthly income, location of residence, hypertension diagnosis, subjective health status, current smoker, and current alcohol use; ^b^ Additionally adjusted for stress.

**Table 3:** Association between mental health status and unhealthy behavior changes among diabetes during the COVID-19.

Characteristics	Stress ^a^			Depression ^b^		
	aOR	95% CI		aOR	95% CI	
		Lower	Upper		Lower	Upper
Changes in physical activity such as walking and exercise						
aOR (95% CI) for decrease	1.35	1.26	1.45	1.34	1.15	1.55
Changes in sleep time						
aOR (95% CI) for decrease	2.21	2.00	2.44	1.87	1.56	2.24
Changes in consumption of junk food or carbonated beverages						
aOR (95% CI) for increase	1.82	1.58	2.09	1.48	1.11	1.99
Changes in food delivery consumption						
aOR (95% CI) for increase	1.50	1.32	1.70	1.54	1.15	2.08
Change in alcohol consumption						
aOR (95% CI) for increase	2.22	1.80	2.72	2.46	1.62	3.71
Change in smoking behavior						
aOR (95% CI) for increase	2.17	1.75	2.70	1.92	1.27	2.90

aOR, adjusted odds ratio; CI, confidence interval; ^a^ Adjusted for sex, age, educational level, family type, monthly income, location of residence, hypertension diagnosis, subjective health status, current smoker, current alcohol use, awareness of glucose level, duration of diabetes, and diabetes treatment; ^b^ Additionally adjusted for stress.

## Data Availability

On request to the corresponding author, all data are available.
